# Impact of* Paracoccidioides brasiliensis* Coinfection on the Evolution of* Schistosoma mansoni-*Induced Granulomatous Liver Injury in Mice

**DOI:** 10.1155/2019/8319465

**Published:** 2019-03-24

**Authors:** Matheus Pereira de Araújo, Eva Burger, Rômulo Dias Novaes, Amanda Ami Akatuti, Maria Ângela Rodrigues, Ana Carolina Silvério Cerqueira Mendes, Giulia Maria de Castro Bani, Eliziária Cardoso Santos, Andréia Aparecida Santos Mendonça, Raquel Lopes Martins Souza

**Affiliations:** ^1^Instituto de Ciências Biomédicas, Universidade Federal de Alfenas (UNIFAL-MG), Alfenas, Minas Gerais, Brazil; ^2^School of Medicine, Universidade Federal dos Vales do Jequitionhonha e Mucuri (UFVJM), Diamantina, Minas Gerais, Brazil

## Abstract

The pathogens* Schistosoma mansoni* and* Paracoccidioides brasiliensis *share common geographic areas, determining infectious diseases with high mortality rates worldwide. Histopathological and immunological changes induced by each pathogen are well understood; however, the host responses to* S. mansoni* and* P. brasiliensis* coinfection are still unknown. Thus, we investigated liver damage and cytokines production in a murine model acutely and chronically coinfected with these pathogens. Fourty male Swiss mice were infected with* S. mansoni* and* P. brasiliensis* alone or coinfected. The animals were euthanized with 50 (acute infection) and 120 (chronic infection) days of infection. All infected animals exhibited liver inflammation. Intense granulomatous inflammation was detected in animals infected with* S. mansoni* alone and those coinfected. Productive and involutive granulomas were clearly observed in acute and chronic infections, respectively. Granuloma size was reduced in the acute phase and increased in the chronic phase of* S. mansoni* and* P. brasiliensis* coinfection, compared with animals infected only with* S. mansoni*. In the chronic phase of infection, the granulomatous inflammation in coinfected animals was characterized by intense neutrophils accumulation and reduced eosinophils number. IFN-*γ*, IL-2, IL-4, and IL-5 circulating levels were increased in all infected groups. Coinfected animals presented attenuated IFN-*γ* and IL-4 production in the acute and chronic infections. Taken together, our findings indicate that coinfected animals exhibited a differential modulation of granulomatous inflammation during the acute and chronic phases of infection, which was potentially associated with a divergent profile of cytokines production and migration of neutrophils and eosinophils in response to* S. mansoni* and* P. brasiliensis *antigenic stimulation.

## 1. Introduction

The development of infectious diseases is deeply influenced by the interaction between pathogen phenotype (i.e., infectivity, pathogenicity, and virulence) and host conditions, such as immunological health and presence of comorbidities, including coinfections [1–3]. Although coinfections are often neglected, these diseases are highly prevalent worldwide, especially in developing countries [4, 5]. Coinfections are more dangerous than infections induced by a single pathogen [3, 6], especially considering that divergent (i.e., cellular* vs.* humoral, or Th1* vs.* Th2* vs.* Th17) and unbalanced immunological phenotypes simultaneously are required to combat two or more parasite species can compromise the infection resolution [3, 7, 8]. In general, coinfections are typically determined by pathogens that share common endemic areas [9], such* Schistosoma mansoni* that causes schistosomiasis in Latin America [10] and the fungus* Paracoccidioides brasiliensis, *the etiological agent of paracoccidioidomycosis [11].

Schistosomiasis is a neglected parasitic disease responsible for more than 230 million people infected in more than 74 countries [10, 12]. In endemic areas, due to systemic manifestations (i.e., granulomatous inflammation and fibrosis in multiple organs of the gastrointestinal, urogenital and respiratory systems), schistosomiasis is responsible for marked morbidity and mortality [10]. In addition,* P. brasiliensis* infects about 10 million people in Latin America [13, 14]. After infection with this thermodimorphic fungus, body temperature of the infected host contributes to transform infective forms (i.e., conidia or mycelial fragments) into its pathogenic forms (yeast-like), which may determine clinical manifestations ranging from asymptomatic infection to severe and disseminated disease [11, 15, 16]. In cases of late treatment or when specific antifungal chemotherapy (i.e., amphotericin and azoles) is not administered, this disease may result in disabling sequelae or death [11, 17]. Although* Schistosoma mansoni* and* P. brasiliensis* share common endemic areas [4, 18], coinfections with these pathogens remains overlooked.

Granulomatous inflammation is the main pathological event associated with severe organ damage in Schistosoma-infected hosts [10, 19]. Granulomatous inflammation is a defense response triggered by antigens of the parasite eggs trapped in host tissues, especially in liver, lung, and spleen [20, 21] The granulomatous inflammation is a dynamic process, which is orchestrated by the host immunological response [20]. In the initial phase of* Schistosoma* oviposition, the formation of schistosomiasis granulomas is mainly mediated by the recruitment of monocytes and neutrophils, which are modulated by Th1 cytokines such as IL-12, INF-*γ*, and TNF-*α* [20]. However, the host develops a typical Th2 response as the infection becomes chronic, determining an intense recruitment of eosinophils and organization of activated macrophages to epithelioid cells [21, 22].

While Th2 phenotype is protective against* Schistosoma* infections [20, 23], Th1 polarization and its typical cytokines (i.e., IL-12, TNF-*α*, and specially IFN-*γ*) are essential to increase host resistance against* P. brasiliensis* [23, 24]. As competitive molecular pathways activate these immunological patterns, it is possible that coinfections with* S. mansoni* and* P. brasiliensis* change the host resistance against both pathogens. Thus, in the course of* Schistosoma*-induced granulomatous inflammation, the attenuation of host defense may favor the opportunistic infection by* P. brasiliensis*. Conversely, it is also possible that* P. brasiliensis* infection change the granulomatous inflammation and organ damage in* Schistosoma*-infected hosts. Considering these hypothesis, by using an experiment model of* S. mansoni* and* P. brasiliensis* coinfection, we investigated if these infections might interact to change cytokines production and organ damage in mice.

## 2. Material and Methods

### 2.1. Animals and Ethics

Forty male Swiss mice were equally randomized in four groups with ten animals in each group: control (CG): uninfected, Sm: infected with* S. mansoni*, Pb: infected with* P. brasiliensis*, and Sm+Pb: infected with* S. mansoni *and* P. brasiliensis. *The animals were maintained in a controlled environment with 60-70% humidity, temperature at 21±2°C, and 12/12h light/dark cycles. Rodent chow and water were provided* ad libitum*. All experimental protocols were approved by the institutional Ethics Committee for Animals Use in Research (protocol 543/2013).

### 2.2. Induction of* S. mansoni* and* P. brasiliensis* Infection

The animals attributed to infected groups were inoculated with the infective forms of the helminth* S. mansoni* and the fungus* P. brasiliensis*. To induce schistosomiasis, mice were subcutaneously infected with 25 cercariae of the LE strain. This strain was obtained from human patients and maintained in multiples passages in* Biomphalaria* snail and Swiss mice [3]. The fungi infection was induced by intraperitoneal inoculation of* P. brasilienses* (virulent Pb18 strain) [25]. The fungi were cultivated in Fava Netto culture medium [26] at 35°C for 7 days. After that time, the cells were washed with 0.85% sterile saline and a fungal suspension was obtained with a concentration of 5 × 10^6^ yeast cells/mL, based on the hemocytometer count. Cell viability was determined by staining of Janus Green B vital dye, and only visualization greater than or equal to 80% of viable cells was used [27]. The animals of each group were intraperitoneally anesthetized (120 mg/kg ketamine and 10 mg/kg xylazine) and euthanized by heart puncture with exsanguination 50 days (acute infection) and 120 days (chronic infection) after inoculation with* S. mansoni* and* P. brasiliensis*.

### 2.3. Confirmation of* S. mansoni* and* P. brasiliensis* Infection

The confirmation of infection was based on the direct microscopic visualization of the etiological agent in the target organs.* S. mansoni* infection was confirmed through histopathological observation of parasite eggs and schistosomiasis granulomas in liver tissue sections stained with hematoxylin and eosin [3].* P. brasiliensis *infection was also confirmed through microscopic observation of mesenteric fungi deposits, which were revealed by the Grocott-Gomori methenamine silver method for detecting fungi [28].

### 2.4. Histopathological and Morphometric Analysis

Liver samples (median lobe) were collected and fixed in fresh 4% paraformaldehyde solution in sodium phosphate buffer (pH = 7.4, 0.1 M). Organ fragments were dehydrated in ethanol, clarified in xylene, and embedded in paraffin. The paraffin blocks were cut in semi-series at 5 *μ*m thick in a rotary microtome and the histological sections were stained with hematoxylin and eosin [29]. In the histopathological analysis, evidence of cell degeneration (hydropic and steatosis), hepatocytes hyper- or hypotrophy, distribution of hemorrhage and inflammatory foci, organization of hepatocytes cords, and evidence of vascular dilatation or collapse were qualitatively described. Parameters such as number, area, and volume of schistosomiasis-elicited granulomas were also measured. The distribution of total leucocytes, neutrophils, and eosinophils was also determined. Granuloma number and size (area and volume) were microscopically determined by using a ×5 (×50 magnification) and ×10 (×100 magnification) objective lens, respectively. Total and differential leucocytes counting were performed with a ×100 objective lens (×1000 magnification). Histological images were captured by using a bright field photomicroscope (Axio Scope A1, Carl Zeiss, German). Ten microscopic images were obtained for each animal and magnification. The images were analyzed and all quantifications were established from the image analysis software AxioVision 4.9.1 (Carl Zeiss, German).

### 2.5. Immunoenzymatic Assay for Cytokines

The impact of the isolated or combined infection with* S. mansoni* and* P. brasiliensis* on cytokines production was investigated from an Enzyme-Linked Immunosorbent Assay (ELISA) sandwich method [30]. Blood samples were collected by cardiac puncture and serum was obtained from centrifugation (5000 ×*g*, 15 min) in the absence of anticoagulant and used to quantify circulating cytokines levels. For the assay procedure, commercial kits were used to detect the cytokines interferon gamma (IFN-*γ*), interleukin-2 (IL-2), interleukin-4 (IL-4), and interleukin-5 (IL-5) according to manufacturer instructions (Peprotech, Ribeirão Preto, Sp, Brazil). Briefly, 96-wells polystyrene plates were incubated with capture antibodies, serum of control, and infected animals. The reaction was developed with a streptavidin-peroxidase conjugated detection antibody (Vector Laboratories, Burlingame, CA, USA) followed by incubation with the chromogen 3,3′,5,5′ tetramethylbenzidine (Promega, Madison, WI, USA). The optical densities (OD) of the samples were detected at 450 nm (Anthos Zenyth 200, Biochrom, Cambridge, UK), and cytokine concentrations were calculated by extrapolating the OD obtained from a standard curve for each recombinant cytokine (SOFTmax PRO 4.0 software, Molecular Devices Corporation, Sunnyvale, CA, USA).

### 2.6. Statistical Analysis

The results were expressed as mean and standard deviation (mean ± SD) or median and interquartile range. Normality in the data distribution was assessed using the Kolmogorov-Smirnov test. The data variance was measured by one-way ANOVA. Parametric data were submitted to the Student-Newman-Keuls* post-hoc* test for multiple comparisons. Nonparametric data were compared using the Kruskal-Wallis test. The results with P value <0.05 were considered statistically significant.

## 3. Results

**Figure 1:**
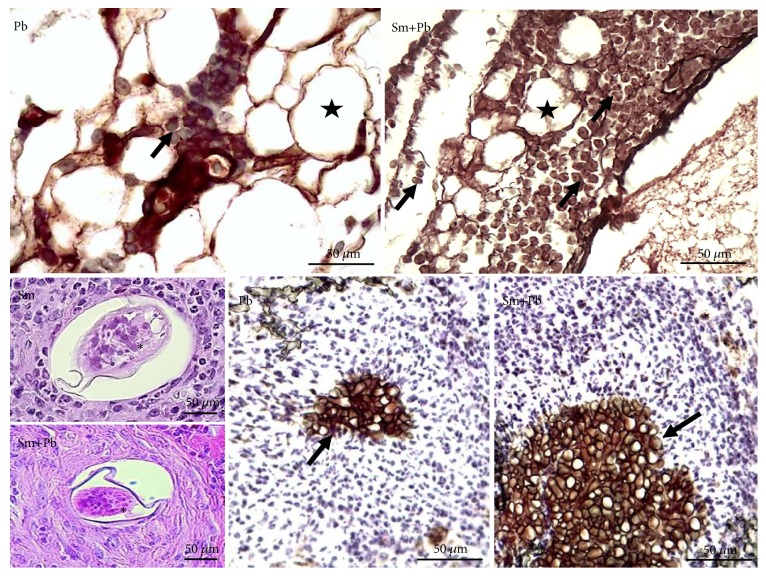
Representative photomicrographs of the liver tissue from control mice and those acutely infected with* Schistosoma mansoni* and* Paracoccidioides brasiliensis*. Pb: group infected with only* P. brasiliensis*. Sm: group infected only with* S. mansoni*. Sm+Pb: group coinfected with* S. mansoni* and* P. brasiliensis*. *∗* * S. mansoni* egg is centrally observed in hepatic granulomas (Hematoxylin and Eosin staining, ×400 magnification, and bars= 50 *μ*m). Adipocytes (stars) and* P. brasiliensis *(yeast-like forms) deposits (arrows) are clearly observed in mesentery (Grocott-Gomori methenamine silver method for fungi with hematoxylin counterstaining, ×400 magnification, and bars= 50 *μ*m).

In all inoculated animals,* S. mansoni* and* P. brasiliensis* infections were confirmed. In* S. mansoni-*infected mice, parasite eggs and hepatic granulomas were clearly observed in bright field microscopy. Similarly, in* P. brasiliensis*-infected animals, yeast-like forms were observed in the mesentery, marked in a dark-brown color by the Grocott-Gomori methenamine silver method. The fungi were clearly defined in the mesentery after hematoxylin counterstaining (Figure 1).

**Figure 2:**
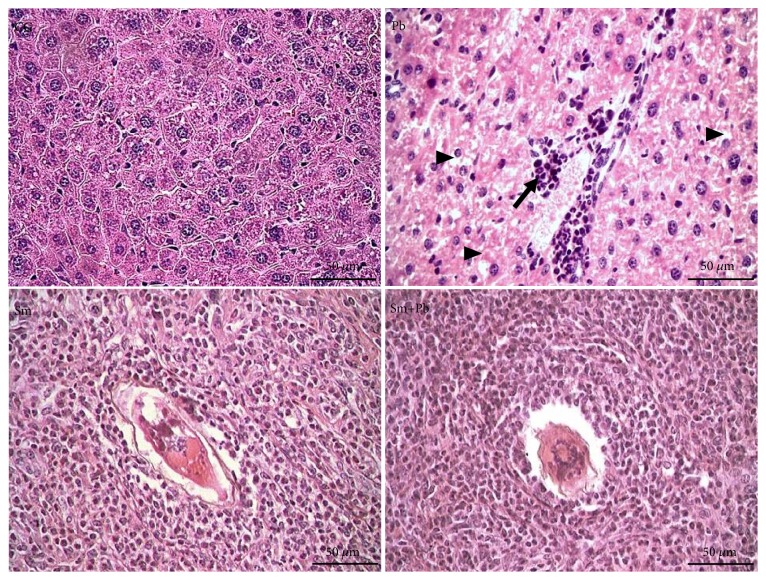
Microscopic structure of the liver tissue from control mice and those acutely infected with* Schistosoma mansoni* and* Paracoccidioides brasiliensis* (Hematoxylin and Eosin staining, ×400 magnification, bars= 50 *μ*m). CG (control group): uninfected mice. Pb: group infected only with* P. brasiliensis*. Sm: group infected only with* S. mansoni*. Sm+Pb: group coinfected with* S. mansoni* and* P. brasiliensis*. A* S. mansoni* egg is centrally observed in granulomas. Arrow: perivascular inflammatory infiltrate. Arrowhead: hepatocytes with hydropic degeneration.

On day 50, time period correspondent to the acute phase of infected groups, the liver of control-uninfected animals exhibited a normal microstructural organization, with regular distribution of hepatocyte cords, well-defined sinusoid capillaries, and reduced interstitial cellularity. Infection with* S. mansoni* and* P. brasiliensis *was confirmed in all animals inoculated with these pathogens. In acute the phase of infection,* S. mansoni* eggs and schistosomiasis granulomas were clearly observed in liver tissue of both infected groups. The intense cellularity indicated granulomatous inflammation in productive stage. In addition to the granuloma sheath, infiltrate leucocytes exhibited pericellular and perivascular distribution, as well as focal inflammatory infiltrate in remote liver areas free of granulomatous reactions. Morphological evidence of hydropic degeneration was more evident in the group infected with* P. brasiliensis *(Figure 2).

**Figure 3:**
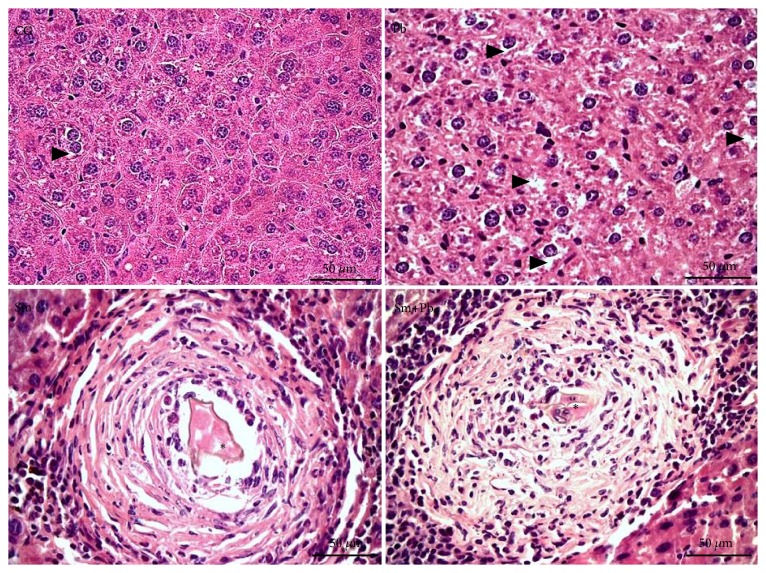
Microscopic structure of the liver tissue from control mice and those chronically infected with* Schistosoma mansoni* and* Paracoccidioides brasiliensis* (Hematoxylin and Eosin staining, ×400 magnification, and bars= 50 *μ*m). CG (control group): uninfected mice. Pb: group infected only with* P. brasiliensis*. Sm: group infected only with* S. mansoni*. Sm+Pb: group coinfected with* S. mansoni* and* P. brasiliensis*. *∗* Sectional profile of* S. mansoni *egg. Arrowhead: hepatocytes with hydropic degeneration.

Control-uninfected mice also exhibited a normal and unchanged liver microstructure in the period correspondent to the chronic phase in infected animals (120 days post-inoculation). Animals infected only with* S. mansoni* and those coinfected with* P. brasiliensis* exhibited a marked change of granuloma organization, which exhibited a clear modulation/involutive pattern. Beyond reduced general cellularity, leucocytes infiltrate was more evident in the outermost areas of the granuloma sheath. The involution phase was characterized by the concentric an epithelioid organization of macrophages around* S. mansoni* eggs and in addition the accumulation of eosinophilic amorphous material in granulomatous sheath (Figure 3).

**Figure 4:**
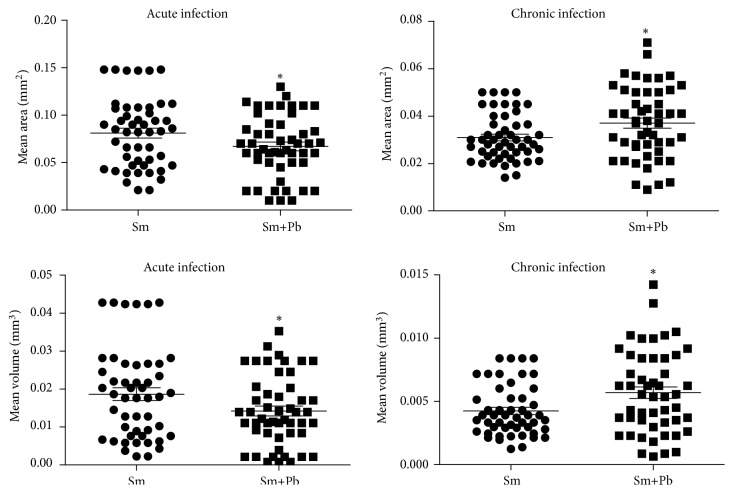
Mean area and volume of schistosomiasis-elicited granulomas in liver from mice acutely and chronically infected only with* Schistosoma mansoni* (Sm) or coinfected with* Paracoccidioides brasiliensis* (Sm+Pb). Data are represented as mean and standard deviation (mean ± SD). The points represent the result of each morphometric count in liver sections obtained from each experimental group. Statistical difference among the groups (*∗*P<0.05).

In the acute infection, granuloma area and volume were higher in animals infected with* S. mansoni* alone compared with animals coinfected with* P. brasiliensis *(P<0.05). Conversely, in chronic infection, granulomas area and volume were higher in animals coinfected with* S. mansoni* and* P. brasiliensis *compared with mice inoculated with* S. mansoni* alone (P<0.05) (Figure 4).

**Figure 5:**
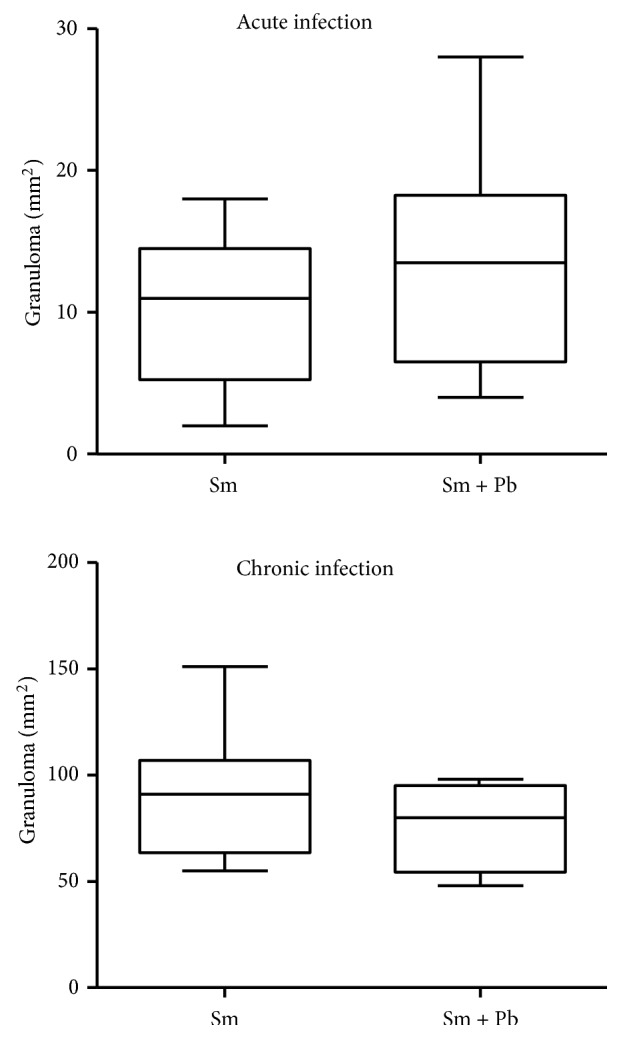
Number density of schistosomiasis-elicited granulomas in liver from mice acutely and chronically infected only with* Schistosoma mansoni* (Sm) or coinfected with* Paracoccidioides brasiliensis* (Sm+Pb). Data are represented as median (central line) and interquartile interval. All groups exhibited similar results (P>0.05).

Despite the differences in area and volume, the number of granulomas was similar in the groups infected with* S. mansoni* and those coinfected with* P. brasiliensis* (P>0.05) (Figure 5).

**Figure 6:**
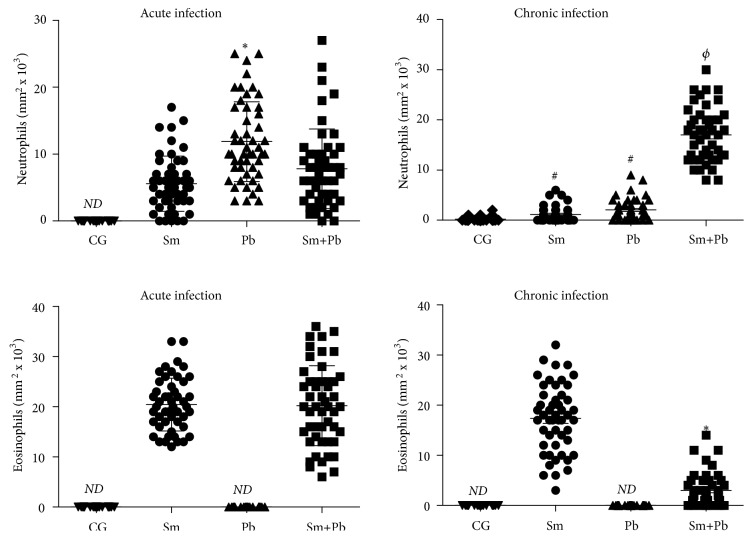
Number density of neutrophils and eosinophils in liver tissue from control uninfected mice (CG) those acutely and chronically infected only with* Schistosoma mansoni* (Sm), only with* Paracoccidioides brasiliensis *(Pb) and coinfected (Sm+Pb).* ND*: nondetected. Data are represented as mean and standard deviation (mean ± SD). The points represent the result of cell counting in liver sections obtained from each experimental group. Statistical difference among groups (P<0.05): *∗vs*. Sm, #*vs*. CG, and Φ*vs*. CG, Sm, and Pb.

During the acute infection, animals infected only with* P. brasiliensis* presented increased number of neutrophils in liver tissue compared with* S. mansoni*-infected mice (P<0.05), which was similar to coinfected animals (P>0.05). Eosinophils infiltrate was similar in animals infected only with* S. mansoni *and those coinfected (P>0.05). These cells were not identified in liver tissue from control animals and those infected only with* P. brasiliensis. *In the chronic infection, animals infected with only* S. mansoni* and only* P. brasiliensis *exhibited similar neutrophils infiltrate, which was markedly increased in coinfected animals (P<0.05). Eosinophils distribution was reduced in coinfected animals compared with mice only infected with* S. mansoni *(P<0.05) (Figure 6).

**Figure 7:**
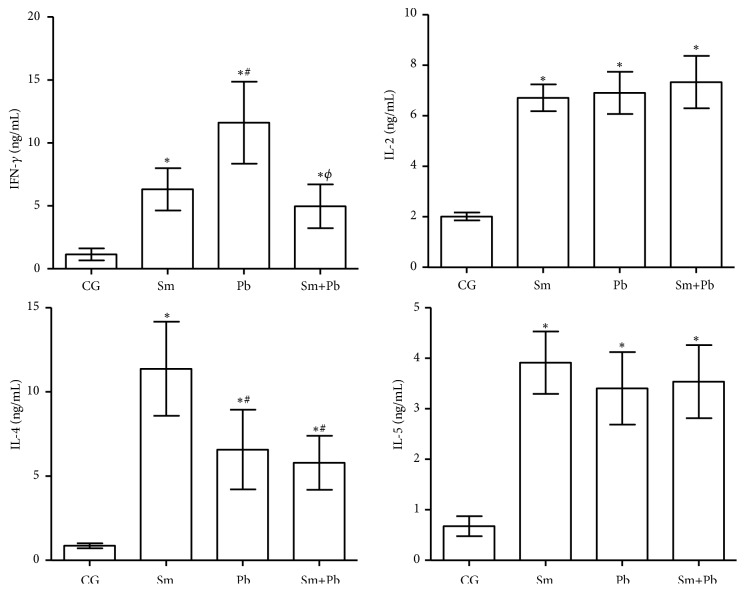
Cytokines serum levels in control uninfected mice (CG) and those acutely infected only with* Schistosoma mansoni* (Sm), only with* Paracoccidioides brasiliensis* (Pb) and coinfected (Sm+Pb). Data are represented as mean and standard deviation (mean ± SD). Statistical difference among groups (P<0.05): *∗vs*. CG, #*vs*. Sm, and Φ*vs*. Pb.

In the acute infection, cytokines serum levels were increased in all infected animals compared with control uninfected mice (P<0.05). Animals infected only with* P. brasiliensis *presented higher IFN-*γ* levels compared with mice infected with only either* S. mansoni *or* P. brasiliensis *(P<0.05), which exhibited similar results (P>0.05). IL-2 and IL-5 serum levels were similar in all infected groups (P>0.05). IL-4 levels were higher in animals infected only with* S. mansoni *compared with mice infected only with* P. brasiliensis* or coinfected (P<0.05), which were similar between them (P>0.05) (Figure 7).

**Figure 8:**
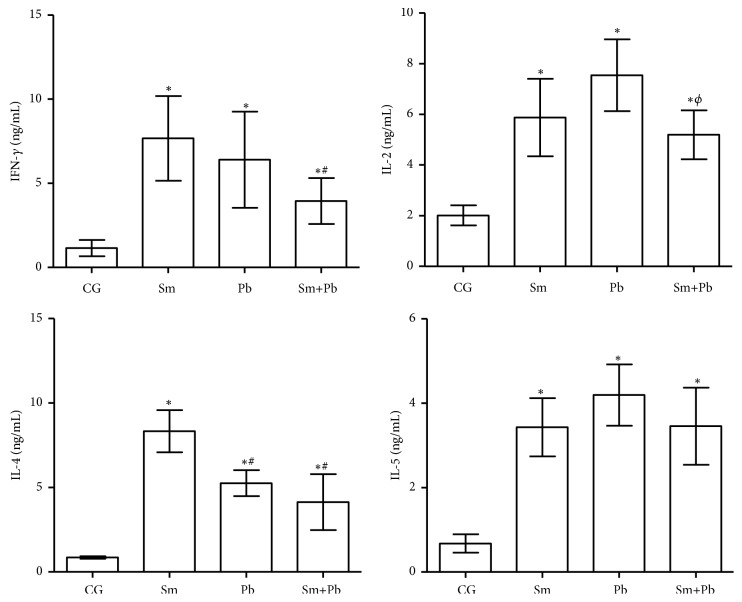
Cytokines serum levels in control uninfected mice (CG) and those chronically infected (120 days after infection) only with* Schistosoma mansoni* (Sm), only with* Paracoccidioides brasiliensis* (Pb) and coinfected (Sm+Pb). Data are represented as mean and standard deviation (mean ± SD). Statistical difference among groups (P<0.05): *∗vs*. CG, #*vs*. Sm, and Φ*vs*. Pb.

In the chronic infection, cytokines serum levels were also increased in all infected animals compared with control uninfected mice (P<0.05). Animals infected with only* S. mansoni *and only* P. brasiliensis *presented similar IFN-*γ* and IL-2 levels (P>0.05). Coinfected mice had reduced IFN-*γ* levels compared with* S. mansoni*-infected animals (P<0.05). IL-2 levels were reduced in coinfected animals compared with mice infected with* P. brasiliensis *alone (P<0.05). IL-4 levels were similarly reduced in animals infected only with* P. brasiliensis *and coinfected compared with mice infected only with* S. mansoni* (P<0.05) (Figure 6). IL-4 serum levels were similar in all infected groups (P>0.05) (Figure 8).

## 4. Discussion

Studies on the interaction between pathogens in neglected infectious diseases are still scarce [31–33]. However, coinfections may be more a rule than an exception in endemic areas, contributing with high rates of morbidity and mortality especially in poor rural communities [3, 34]. According to Coley et al. [10], the increased risk of individuals with schistosomiasis in developing infections by opportunistic microorganisms as fungi is potentially related to the immunoregulatory response established during the clinical course of schistosomiasis (i.e., Th-2 suppressive pattern). From our experimental protocol, it was possible to develop a consistent model of* S. mansoni* and* P. brasiliensis* coinfection. Thus, we clearly observed the coexistence of* S. mansoni* eggs in liver and yeast-like forms of* P. brasilienses* in the mesentery of inoculated animals.

According to Lenzi et al. [35] and to Hams et al. [36], the granulomas of* S. mansoni*-infected mice represent the most striking histopathological feature in the course of infection. In addition, granuloma organization can be classified in different evolutionary stages such as pregranulomatous (i.e., reactive or exudative), granulomatous (exudative-productive or productive), or involutive (with dissociation of collagen fibers) [35]. From a microstructural analysis, our findings were consistent with two well-defined evolutionary stage described by Lenzi et al. [35] and Hams et al. [36]. In the acute infection, intense inflammatory infiltrate and accumulation of mononuclear and polymorphonuclear leucocytes in a thick inflammatory sheath around* S. mansoni* eggs were the main characteristic of productive granulomas. In the chronic infection, modulation/involutive granulomas were typically characterized by a granulomatous sheath with reduced cellularity, marked deposition of eosinophilic collagenous matrix, and dissociated collagen fibers.

Considering the dynamism of leucocytes migration and cellular organization in* S. mansoni*-induced granulomas in acute infections, Amaral et al. [37] described a more pronounced response associated with the occurrence of necrotic-exudative granulomas (~80%) in the liver during the acute phase of* S. mansoni* infection in mice, indicating an exacerbated inflammatory response induced by antigens of this trematode. Apparently, in our study this response pattern was not impaired when the animals were coinfected with* P. brasiliensis*. However, different models of coinfection between* S. mansoni* and HIV,* Plasmodium* sp., and* Mycobacterium tuberculosis* indicated a more intense leucocytes recruitment and inflammatory liver damage in the acute stage of granuloma formation [3, 10, 38]. Conversely, when the prolonged infection (120 days) was investigated, the histopathological analyses in animals infected only with* S. mansoni* and those coinfected indicated modulation/involutive granulomas exhibiting concentric an epithelioid organization of macrophages around* S. mansoni* eggs. Considering the poor clinical outcome of* S. mansoni*-infected hosts in combating coinfecting pathogens that induces divergent immunological patterns (i.e., Th1) [39–41], we expected that the fungi* P. brasiliensis* could modify the cellular pattern of the granulomatous reaction. Such modifications were observed by Farah et al. [42], who investigated a coinfection model of* S. mansoni* and* Papio cynocephalus anubis* in BALB/c mice. In the coinfection course, these authors reported hepatic fibrosis associated with the development of multinucleated giant cells (MGCs), divergent phenotypic profile of inflammatory cells, intense collagenogenesis, and periportal fibrosis. In our study, the coinfection of schistosomiasis animals with* P. brasilienses* did not exhibit the same manifestations expected for* P. cynocephalus anubis *coinfection. According to Almeida et al. [43], although* P. brasiliensis* may infect and develop in multiple organs, the disease often presents a self-limited course or remain asymptomatic for years. In this context, our experimental model was consistent with the human infection, in which infected hosts can exhibit continuous antigenic stimulation in a long-term infectious processes, developing low-grade immunological responses previously to the occurrence of clinical manifestation of paracoccidioidomycosis [44].

In our investigation, the area and volume of schistosomiasis-elicited granulomas in the acute phase of infection were proportionally larger compared with coinfected animals, suggesting a potential association of this fungus with an attenuated granulomatous inflammation. According to Lenzi [35], the intense leukocytes aggregation in the productive phase in animals infected with* S. mansoni* alone is coherent with the formation of large granulomas. However, in coinfected animals the reduction in granuloma size could reflect an adaptation of the immunological response to simultaneously combat different pathogens. Considering that the target organs of both parasites investigated are different, it is not unrealistic to assume that in coinfection the simultaneous recruitment of leucocytes to different sites can attenuate the immune response directed to a single pathogen [45]. This proposition could be mainly expected in acute infections, in which the host immune system is still not adapted to combat different pathogens simultaneously [3].

Compared to the acute stage, in the chronic infection the granulomatous response behaved in an opposite way. Thus, animals infected with* S. mansoni* alone presented a more pronounced granuloma involution, a response similar to those observed in a previous study of* S. mansoni* infection [37]. However, in the chronic infection, coinfected animals exhibited larger granulomas than mice infected with* S. mansoni* alone. This finding seems to be potentially aligned with our proposition of a latency period, which would be required for the immune system to recognize and adapt in response to multiples antigenic stimuli, establishing a more pronounced inflammatory response. This proposition is reinforced considering the morphological, molecular, and functional changes in the infective yeast forms of* P. brasiliensis* after host infection [43, 46]. These changes are an essential adaptive strategy of the fungus, which is important to ensure a proper adaptation to the microenvironment of infected organs [43, 46]. In this process, changes in molecular profile in different evolutionary forms of* P. brasiliensis* creates a latency period, in which the immune system activates variable defense mechanisms to improve antimicrobial responses [43, 46]. Thus, this differential granulomatous process observed in the transition from the acute to the chronic phase of infection may represent a morphological evidence of this latency period. Despite this differential profile related to the size of schistosomiasis granuloma, the number of granulomas remains unchanged. We observed the number of granulomas can increase or decrease according to several parameters, such as host susceptibility (i.e., animal lineage), parasite strain (i.e., pathogens with divergent profiles of infectivity, virulence and pathogenicity), the dynamics of ovulation/oviposition by* S. mansoni* female, and the level of egg retention in host tissues [35, 36].

The size and distribution of schistosomiasis granulomas have been intimately linked to the profile of leucocytes migration [37]. According to Lenzi [35], the composition of schistosomiasis granulomas is characterized by the presence of polymorphonuclear and mononuclear cells, specially eosinophils, macrophages, lymphocytes, neutrophils, mast cells and fibroblasts. Considering animals infected with a single pathogen, our findings indicated that the number of neutrophils was predominantly higher in the acute infection with* P. brasiliensis* as compared to* S. mansoni* Infection. Conversely, the distribution of these cells in schistosomiasis granulomas was markedly reduced in the chronic infection. The high quantity of these cells in the acute infection was already expected, since the onset of the protective immunological response is notably mediated by neutrophils. However, in chronic infections, the granuloma presented a differential stage of evolution, and these cells become less relevant to combat this pathogen [47]. Although these are typical characteristics in monoinfections with* P. brasiliensis*, in the chronic phase of infection, we observed intense neutrophilia in coinfected animals, a process potentially related to the control of fungi dissemination in hepatic tissue.

In our study, the granulomas induced by* S. mansoni* eggs also presented high eosinophilia in the acute and chronic infection. In acute infections, eosinophils constitute approximately 90% of the total cells in granuloma sheath [48]. According to Wynn et al. [49], the increased distribution of these cells may occur before eggs deposition begins. This occurs due to their functions aimed at stimulating antibody-dependent cellular cytotoxicity in different stages of the parasite cycle [50, 51]. Interestingly, granulomatous lesions in coinfected mice also exhibited a predominance of eosinophils in the acute infection. However, in the chronic phase,* P. brasiliensis *coinfection contributed to the drastic reduction of these cells in detriment of the neutrophils predominance in granuloma sheath. According to Chuah et al. [23], in the initial stages of development, the schistosomiasis granuloma is mainly characterized by mononuclear cells, neutrophils, and eosinophils accumulation around the newly deposited egg. Thus, this process can contribute to the formation of a neutrophilic microabscess characterized by a productive-exudative process [23].

According to Chuah et al. [23], the higher or lower ratio between neutrophils and eosinophils that mark the variations in cellular composition of schistosomiasis granulomatous is accompanied by a differential Th1 and Th2 immunological pattern during the course of infection. Several studies have pointed the predominance of proinflammatory Th1 cytokines such as IFN- *γ*, TNF-*α*, IL1, IL2, and IL-5 stimulated by antigens of schistosomules and adult worms present in liver and mesenteric veins of the infected hosts [36, 47, 52]. However, our results showed a divergent response, with higher IFN-*γ* levels in animals infected with* P. brasiliensis* compared with* S. mansoni* alone. Calich et al. [53] showed that the host resistance against* P. brasiliensis* is in fact linked to the predominance of IFN-*γ* production by Th1 lymphocytes, while the susceptibility is mainly associated with an impaired cellular immune responses and activation of B cells [53].

In the chronic phase, both groups infected with a single pathogen maintained high IFN-*γ* levels. Classically, the granuloma evolution is related to a Th2 cytokine profile (i.e., IL-4, IL-5, and IL-13) and lower IFN-*γ* expression [23]. It is possible that this differential pattern with increased IFN-*γ* levels will reflect the cellular reorganization of the schistosomiasis granulomatous as a reflex of the beginning of an involutive granulomatous phase. On the other hand, when coinfected animals are analyzed in both acute and chronic phases, similar IFN-*γ* levels were observed, indicating the predominance of an immunological pattern associated with the resistance against* P. brasiliensis* infection [54]. This response profile is divergent from those observed in coinfections involving* S. mansoni* and other pathogens such as* T. cruzi*,* Fasciola hepatica*, and* Heligmosomoides polygyrus* [3, 7, 55], which pointed that* S. mansoni *infection increased the host susceptibility to coinfecting pathogens.

Unlike IFN-*γ*, we observed similar IL-2 and IL-5 levels in the acute and chronic phases in both infected groups. As important proinflammatory cytokines involved in leucocytes recruitment directed to the initial granuloma formation (Stadecker 1999), such high values in the acute phase of* S. mansoni* infection are expected. Although reduced IL-2 levels are reported in chronic infections in response to Th2 polarization [47], antigens of adult worms can continuously stimulate the production of these cytokines, an aspect related to the high IL-2 and IL-5 levels in the chronic phase of* S. mansoni* infection. In addition, in animals inoculated with* P. brasiliensis *alone and those coinfected, sustained production of Th1 cytokines (i.e., IL-2 and IL-5) in both phases of infection can also indicate a protective response associated with the resistance of infected hosts against disease progression [3, 56].

High IL-4 serum levels in the acute and chronic phases in both infected groups were also observed. However, animals infected with* P. brasiliensis* alone and those coinfected exhibited higher IL-4 levels than mice infected with* S. mansoni* alone. This interesting find corroborates the evidence that Th2 cytokines are also produced in infections by pathogens that induces strong Th1 responses, such as* P. brasiliensis* [53] and* T. cruzi* [3]. Since unbalanced immunological responses are dangerous to the host [3, 47], an adequate Th1 and Th2 balance is essential to limit parasite progression without induce extensive damage to health host cells [53]. As observed in our study, high IL-4 levels are expected in* S. mansoni* infection, being used a complementary marker of disease chronification [47]. As* P. brasiliensis *predominantly induces a Th1 immunological polarization [53], it was not surprisingly that coinfected animals presented attenuated IL-4 levels compared with mice infected with* S. mansoni *alone. IL-4 also exerts a direct impact on the evolutionary behavior of the granulomatous infection [47]. Thus, the variations in IL-4 levels could be partially associated with the cellular composition of granulomatous lesions and the divergent profiles of granuloma involution in animals infected with a single parasite and those coinfected.

## 5. Conclusions

Taken together, our findings indicate that coinfected animals exhibited a differential modulation of granulomatous inflammation in the acute and chronic phases of infection, which was potentially associated with a divergent profile of cytokines such as IFN-*γ*, IL-2, IL-4, and IL-5, as well as distinct recruitment of neutrophils and eosinophils in response to* S. mansoni* and* P. brasiliensis *antigenic stimulation. As we have not identified any studies investigating coinfections by these pathogens, further studies are required and urgent. To investigate new models of coinfection from different animal species and lineages as well as* S. mansoni* and* P. brasiliensis* strains with distinct virulence and pathogenicity phenotypes may improve the understanding of the immune response profiles associated with the host resistance or susceptibility to coinfections by these pathogens.

## Data Availability

The data used to support the findings of this study are included within the article.
